# Male Gender is not a Risk Factor for the Outcome of Laparoscopic Cholecystectomy: A Single Surgeon Experience

**DOI:** 10.4103/1319-3767.39622

**Published:** 2008-04

**Authors:** Abdulmohsen A. Al-Mulhim

**Affiliations:** Department of Surgery, King Fahd Hospital of the University, Al-Khobar, Saudi Arabia

**Keywords:** Acute cholecystitis, complications, conversion, experience, gender, laparoscopic cholecystectomy

## Abstract

**Background/Aim:**

Previous studies regarding the outcome of laparoscopic cholecystectomy (LC) in men have reported inconsistent findings. We conducted this prospective study to test the hypothesis that the outcome of LC is worse in men than women.

**Materials and Methods:**

Between 1997 and 2002, a total of 391 consecutive LCs were performed by a single surgeon at King Fahd Hospital of the University. We collected and analyzed data including age, gender, body mass index (kg/m^2^), the American Society of Anesthesiologists (ASA) class, mode of admission (elective or emergency), indication for LC (chronic or acute cholecystitis [AC]), comorbid disease, previous abdominal surgery, conversion to open cholecystectomy, complications, operation time, and length of postoperative hospital stay.

**Results:**

Bivariate analysis showed that both genders were matched for age, ASA class and mode of admission. The incidences of AC (*P* = 0.003) and comorbid disease (*P* = 0.031) were significantly higher in men. Women were significantly more obese than men (*P* < 0.001) and had a higher incidence of previous abdominal surgery (*P* = 0.017). There were no statistical differences between genders with regard to rates of conversion (*P* = 0.372) and complications (*P* = 0.647) and operation time (*P* = 0.063). The postoperative stay was significantly longer in men than women (*P* = 0.001). Logistic regression analysis showed that male gender was not an independent predictor of conversion (Odds ratio [OR] = 0.37 and *P* = 0.43) or complications (OR = 0.42, *P* = 0.42). Linear regression analysis showed that male gender was not an independent predictor of the operation time, but was associated with a longer postoperative stay (*P* = 0.02).

**Conclusion:**

Male gender is not an independent risk factor for satisfactory outcome of LC in the experience of a single surgeon.

Laparoscopic cholecystectomy (LC), first performed by Mühe in 1985,[1] has rapidly become the standard treatment for symptomatic gallstones.[2,3] Advantages of LC over the conventional technique include less postoperative pain, less impairment of vital functions, shorter hospital stay, rapid return to normal activity and work, fewer complications, better cosmesis, and lower cost.[3–7]

Several clinical and epidemiological studies suggest that the outcome of LC depends on factors such as age, gender, body weight, clinical presentation, previous abdominal surgery, and surgeon's experience.[3,8–23] Men tend to present with more severe gallstone disease than women.[22–24] Hence, male gender has been cited by several studies as a risk factor for conversion and complications of LC.[8–10,12–16,19–23] Other studies have failed to identify male gender as a risk factor for the outcome of LC.[17,25–28] Indeed, one study showed that female gender was a distinct risk factor for major bile duct injuries during LC.[29]

Laparoscopic cholecystectomy at most hospitals is generally a collective experience of a team of surgeons. The experience of multiple surgeons is often a major confounder in studies examining preoperative factors as predictors of the outcome of LC. This study analyzed the data of a large cohort operated by a single surgeon. The aim was to test the hypothesis that LC in men is associated with increased rates of conversion and complications, as well as longer operation time and postoperative hospital stay.

## MATERIALS AND METHODS

This prospective study comprised a consecutive cohort of LCs performed by the author at King Fahd Hospital of the University (KFHU) between 1997 and 2002. All procedures were performed under general anesthesia using a standard four-port technique. Pneumoperitoneum was created using Veress needles, except in a few patients who had undergone previous upper abdominal surgery in whom the open (Hasson) technique was used.

The collected data included age, gender, body mass index (kg/m^2^), American Society of Anesthesiologists (ASA) class, mode of admission (elective or emergency), indication for LC, co-morbid disease, and previous abdominal surgery. The outcome measures for the study were conversion to open cholecystectomy (OC), operation time, length of postoperative hospital stay, and complications detected during the same admission or over a period of 12 weeks after discharge. Based on the body mass index (BMI), patients were classified as nonobese (BMI < 30), obese (BMI ≥ 30 to < 40) and morbidly obese (BMI ≥40 or ≥35 along with one of the comorbidities such as cardiovascular disease, hypertension, Type II diabetes, osteoarthritis, infertility, sleep apnoea, and ovarian/endometrial cancer). Clinically, patients were classified into two sub-groups: biliary colic-chronic cholecystitis (“chronic”) and acute cholecystitis (“acute”). The diagnosis of acute cholecystitis (AC) was based on typical clinical, ultrasound, operative, and pathological findings.[30] Patients with obstructive jaundice and pancreatitis due to stones in the common bile duct (CBD) underwent endoscopic sphincterotomy and stone clearance before LC. They were then classified as chronic or acute subgroup based on their gallstone symptoms. With regard to biliary duct injuries, only those requiring endoscopic or surgical intervention were included. Minor bile leak cases treated with drainage or drains left ***in situ*** after primary surgery were excluded. The author personally verified and validated the completeness of the data entered into the computer.

### Statistics

Data were analyzed using SPSS version 11.5 for Windows. Descriptive statistics (mean and standard deviation) were used for quantitative variables. Bivariate analyses using ***t***-test for continuous parametric variables and chi-square (χ^2^) test for nonparametric variables were performed. Multiple logistic regression analysis for dichotomous variables (conversion and complications of LC) and linear regression model for continuous variables (operation time and length of postoperative stay) were employed. Results are presented as odds ratio (OR) with 95% confidence interval (CI). Two-sided significance with ***P*** < 0.05 is reported.

## RESULTS

### Overall results

During the study period, LC was attempted in 391 patients (282 [72%] females) with a mean age of 38.8 years. The mean BMI was 28.9, over 90% of the patients were ASA class I or II, 18% were admitted as emergencies and 15% had AC. Comorbid diseases were found in 35.6% of the patients and previous abdominal surgery was present in 26.2%.

No major complications were encountered during creation of pneumoperitoneum or cholecystectomy in either obese patients or those who had previous abdominal surgery. Seven patients (1.8%) required conversion to OC. The causes for conversion were obscure anatomy in five patients, and liver bleeding and equipment failure in one case each.

The complication rate was 3.8% and included atelectasis in three patients; wound infection, urinary tract infection, chest infection, epigastric port site hernia (two each); and wound hematoma, massive liver bleeding, CBD injury, and major cystic duct injury (one each). Postoperative surgical intervention was required in patients with CBD and cystic duct injuries, and port site hernias. There was no mortality in this series. Average operation time, including converted cases, was 75.2 minutes. The mean postoperative stay was 2.6 days for all patients.

### Bivariate analysis

Table 1 shows bivariate analysis of preoperative characteristics by gender. There were no statistical differences between genders in terms of age (***P*** = 0.063), ASA class (***P*** = 0.338), and mode of admission (***P*** = 0.069). The incidences of AC (***P*** = 0.003) and co-morbid disease (***P*** = 0.031) were significantly higher in men than women. Hypertension and diabetes mellitus were the most common comorbidities in women and men, respectively. Women were significantly more obese than men (***P*** < 0.001) and had a higher incidence of previous abdominal surgery (***P*** = 0.017) with appendectomy and cesarean section accounting for 49% of the operations.

**Table 1:** Laparoscopic cholecystectomy (LC): Characteristics of 391 patients

Data	Men (*n* = 109)	Women (*n* = 282)	*P* value
Age (mean ± SD), years	40.8 ± 14.7	38 ± 12.5	0.063
BMI (mean ± SD)	26.6 ± 5.1	29.9 ± 5.9	<0.001
ASA class	109	282	0.338
I	44 (40.4)	110 (39.0)	
II	61 (56.0)	150 (53.2)	
III	4 (3.7)	22 (7.8)	
Mode of admission	109	282	0.069
Elective	83 (76.1)	237 (84.0)	
Emergency	26 (23.9)	45 (16.0)	
Indication for LC	109	282	0.003
CC	83 (76.1)	250 (88.6)	
AC	26 (23.9)	32 (11.4).	
Comorbld disease	48 (44.0)	91 (32.6)	0.031
Diabetes mellitus	14	22	
Cardiovascular	7	24	
Respiratory	3	7	
Sickle cell disease	5	6	
Others	19	32	
Previous abdominal	19 (17.6)	83 (29.4)	0.017
surgery			
Appendectomy	8	15	
Cesarean section	0	27	
Others	11	41	

SD: Standard deviation, BMI: Body mass index (kg/m^2^), ASA: American Society of Anesthesiologists, CC: Chronic cholecystitis, AC: Acute cholecystitis, Values in parentheses are percentages.

Table 2 shows that there were no statistical differences between genders with regard to conversion to OC (***P*** = 0.372), operation time (***P*** = 0.063), and complications (***P*** = 0.647); however, the postoperative stay was significantly longer in men than women (***P*** = 0.001).

**Table 2:** Outcome of laparoscopic cholecystectomy in 391 patients

Data	Men (*n* = 109)	Women (*n* = 282)	*P* value
Conversion	3 (2.8)	4 (1.4)	0.372
Operation time (mean ± SD), min	80.3 ± 51.2	70.4 ± 44.6	0.063
Morbidity	5 (4.6)	10 (3.6)	0.647
Post-operative stay (mean ± SD), days	3.2 ± 3.1	2.4 ± 1.8	0.001

SD: Standard deviation, Values in parentheses are percentages.

### Multivariate analysis

Multivariate logistic regression analysis demonstrated that none of the tested variables, including male gender, was an independent predictor of conversion to OC [Table 3] or complications [Table 4]. Although conversion to OC was almost eight times more common in emergency than elective admissions (OR = 7.9), the difference was not statistically significant (***P*** = 0.07).

**Table 3:** Multivariate analysis for risk of conversion of laparoscopic to open cholecystectomy in 391 patients

Data	Odds ratio	95% Cl	*P* value
Gender			
Male	0.37	0.03-4.24	0.43
Female	1.0	-	-
Age (continuous)	1.04	0.97-1.11	0.23
BMI (continuous)	0.91	0.77-1.08	0.29
ASA class			
III	0.98	0.56-567.92	0.98
I/II	1.0	-	-
Admission			
Emergency	7.91	0.87-71.79	0.07
Elective	1.0	-	-
Indication for operation			
AC	1.1	0.86-14.08	0.94
CC	1.0	-	-
Comorbid disease			
Yes	1.47	0.22-9.67	0.69
No	1.0	-	-
Previous abdominal surgery			
Yes	1.33	0.142-12.18	0.8
No	1.0	-	-

Cl: Confidence interval, BMI: Body mass index (kg/m^2^), ASA: American Society of Anesthesiologists, CC: Chronic cholecystitis, AC: Acute cholecystitis

**Table 4:** Multivariate analysis for risk of complications of laparoscopic cholecystectomy in 391 patients

Data	Odds ratio	95% Cl	*P* value
Gender			
Male	0.42	0.03-4.24	0.42
Female	1.0	-	-
Age (continuous)	1.01	0.97-1.11	0.77
BMI (continuous)	1.07	0.77-1.08	0.26
ASA class			
III	0.00	0.56-567.92	0.99
I/II	1.0	-	-
Admission			
Emergency	0.35	0.87-71.79	0.40
Elective	1.0	-	-
Indication for operation			
AC	2.52	0.86-14.08	0.33
CC	1.0	-	-
Comorbid disease			
Yes	0.40	0.22-9.67	0.29
No	1.0	-	-
Previous abdominal surgery			
Yes	0.29	0.142-12.18	0.27
No	1.0	-	-

Cl: Confidence interval, BMI: Body mass index (kg/m^2^), ASA: American Society of Anesthesiologists, CC: Chronic cholecystitis, AC: Acute cholecystitis

Multiple linear regression analysis demonstrated that age (***P*** = 0.03) and AC (***P*** = 0.001) were independent predictors of operation time. Male gender (***P*** = 0.29), BMI (***P*** = 0.07), ASA class (***P*** = 0.28), mode of admission (***P*** = 0.83), previous abdominal surgery (***P*** = 0.65), and comorbid disease (***P*** = 0.93) were not independent predictors of the operation time. Male gender (***P*** = 0.02) was the only independent predictor of prolonged postoperative stay. Age, BMI, ASA class, mode of admission, indication for LC, comorbid disease, and previous abdominal surgery did not predict length of postoperative stay.

## DISCUSSION

Cholecystectomy is the only operation in which laparoscopy is the gold standard.[3] The benefits of LC to the patient and community are well documented. [3–7] Initially, contraindications for LC included AC, obesity, previous abdominal surgery, CBD stones, gallstone pancreatitis, and liver cirrhosis.[4,5] With increasing experience, patients with morbid obesity and complicated AC are no longer excluded.[11,18]

### Conversion

Since the introduction of LC at KFHU, the procedure was offered to all patients with symptomatic gallstones. The findings reported herein indicate that this policy is not associated with increased rate of conversion to OC. The 1.8% overall conversion rate among the study patients is similar to that reported by Bittner[3] but less than the 5–19% reported by others.[8–17,20,21,25,26,28] Several factors may contribute to this finding. First, patients in this series were relatively younger than those in other studies.[4,7,9,10,14–16,20,21,26] Elderly patients (≥60 years) are a risk group for late presentation, complicated gallstones, and comorbid disease.[18,24,31] Several studies have documented increased rates of conversion, morbidity and mortality, and prolonged operation time and hospital stay after LC in the elderly.[8,10,14,16,18–22,25,26,31] In this series, the mean age of the patients was 39 years and 90% were below the age of 60 years, which excludes age as a risk factor. Second, most of our patients were nonobese rendering the procedure technically easier with a significantly shorter operation time.[11] Third, all LCs in this series were performed by a single surgeon with experience in laparoscopic surgery, who used a standardized approach, testifying that the surgeon's experience does influence the rate of conversion to OC.[3,20]

The finding of similar rate of conversion for both genders was somewhat surprising, especially that men in this series had a significantly higher incidence of AC (***P*** = 0.003). This finding is indeed at variance with others[18] relating to our practice of offering early LC to patients with AC when the dissection is relatively easier, and anatomy more discernable.[30]

It is still controversial whether conversion to OC is really a “complication” of LC. While the contrary argument of labeling conversion as a “complication”[32] must be respected, we, like many others,[4,5,14,16] consider conversion a safe strategy to prevent serious complications during LC. We, therefore, instruct the trainee residents to have a low threshold for conversion to open surgery throughout the procedure. In agreement with others, the most common cause for conversion in our cohort was difficulty in delineating the anatomy at Calot's triangle.[8,10,13,16,17,20]

### Complications

The overall rate of complications (3.8%) in this series is similar to that found in the current literature,[3,10,20,21] and most of these were successfully managed by nonsurgical measures. Although intraoperative cholangiogram (IOC) was not performed in any patient in this series, the CBD injury rate of 0.3% compares favorably to that reported by others.[3,4,7,20,21,33] We believe that IOC should not be performed routinely as it does not decrease the risk of biliary tree injury[34] particularly now that accurate and less invasive assessment of CBD pathology can be made preoperatively by magnetic resonance cholangiopancreatography.[35]

### BMI

Obesity was considered a contraindication to LC,[4] because abdominal surgery in the obese is difficult with increased risk of complications. Although the BMI was significantly different in men and women (***P*** < 0.001), it had no influence on the outcome of LC. This is probably because most of the patients in this study were nonobese (mean BMI = 29). This finding is similar to other studies that failed to show a significant influence of BMI on the conversion and complications of LC,[11] even in patients with BMI > 30.[36] Hutchinson ***et al.***[9] studying 526 patients, however, showed that BMI > 27.2 was a risk factor for conversion. Despite this variance in the literature, we like Miles ***et al.***[37] of the opinion that laparoscopy should be the preferred method of cholecystectomy in obese patients in whom early mobilization and shorter postoperative stay reduce the incidence of complications.

### Acute cholecystitis

Laparoscopic cholecystectomy for AC is technically demanding and may take longer time to perform, yet it is safe in the hands of experienced surgeons.[3,30] Nevertheless, there is still debate on the optimum surgical approach and the timing of surgical intervention. A recent Swiss study concluded that early open and laparoscopic techniques had similar morbidity rates and no mortality, but LC was associated with a significantly longer operation time and a shorter postoperative hospital stay.[38] Prospective randomized studies comparing early with delayed LC for AC have shown no significant differences in the conversion rate, operation time and postoperative complications either,[39–41] yet early LC is associated with a significantly shorter hospital stay and lower costs.[41] Ishizaki ***et al.***[17] attributed the increased rate (0.9%) of bile duct injury in their series to the 2–4 weeks delay in performing LC for AC. Overall, the balance seems to tip in favor of an early LC for an acute case, preferably within the “golden 72 hours” of admission as was the practice with the early open technique.

### Previous surgery

In patients with previous abdominal surgery, the surgeon may experience difficulty during insertion of the Veress needle or the first trocar. Varying degrees of adhesiolysis may be required to adequately expose the operative field. We used the blind technique to create pneumoperitoneum and insert the first trocar in most of the patients, including those with previous abdominal surgery. This approach was safe with no vascular or visceral injury.

In this study, previous abdominal surgery was more prevalent in women than men (***P*** = 0.017), but was not a risk factor for conversion, complications, operation time, or postoperative stay in either gender. This is probably because most of the patients with previous abdominal surgery had procedures in the lower abdomen leaving the upper part fairly free of adhesions. Karayiannakis ***et al.***[42] documented increased rates of conversion and postoperative wound infection, and longer operation time and postoperative stay after LC for patients with previous upper abdominal surgery than those with lower or no previous abdominal surgery.

### Operation time

Our overall mean operation time (75 min) is similar to that of other series.[4,7] Although the mean time was longer in men than women (80.3 vs. 70.4 min), it was not statistically significant (***P*** = 0.063). On multivariate analysis, age (***P*** = 0.03) and AC (***P*** = 0.001) were found to be independent predictors of the operation time. Gender, BMI, ASA class, mode of admission, previous abdominal surgery, and comorbid disease did not influence the operation time. This finding is relevant now that most of the LCs are projected to be day-case procedures.[35,43–45]

### Postoperative stay

In this study, the postoperative stay was significantly longer for men (***P*** = 0.001). This is probably attributed to the increased rate of comorbid disease in men than women (***P*** = 0.031) requiring extended medical management after surgery. The patients can thus be informed that in view of their co-existent medical conditions their postoperative stay is likely to be longer and they are unlikely to be scheduled as a day-case LC. This is relevant to strategies in utilization of operating room schedule as well as preparation of patient information material.

### Surgeon's experience

For surgeons experienced in conventional laparotomy, LC may be more challenging to master than younger surgeons trained from the outset in minimal invasive surgery. That aspect of training is however, not a dominant factor for safe LC. It is the overall experience in the technique ***per se***, as evidenced by several reports that the risk of conversion and complications of LC are inversely related to the total experience of a particular surgeon.[3,5,10,20,21,22,46] However, it is also true that experienced surgeons may have increased conversion rates, because they operate on complex cases.[47] The CBD injury reported here did not occur among the first 100 LCs performed “during the learning curve.” This may be attributed to the surgeon's over confidence and reluctance for conversion to OC.

### Gender

The results of this study indicate that male gender is not a significant predictor of the outcome of LC. Few reports have shown that male gender is not a risk factor for conversion to OC.[17,25–28] Although Gronroos ***et al.***[29] reported that women were at a higher risk for severe bile duct injuries during LC, most studies have shown that the outcome of LC is worse in men.[8–10,12–16,19–23] This likely relates to the increased severity of gallstone disease in men.[22–24] In this study, men accounted for 28% of the patient population, but 44.9% of those presented with AC (***P*** = 0.003).

A structured analysis of the current literature shows that AC, but not the male gender, is the strongest significant risk factor for conversion [Table 5] and complications of LC.[21] In addition, risk factors, such as abdominal pain and tenderness, leucocytosis and ultrasound finding of a thick-walled gallbladder, are related to AC.[8,9,12,14,17,26–28] Similar to Ibrahim ***et al.***,[20] we were unable to determine the effect of preoperative ultrasound findings, such as gallbladder wall thickness,[8,9,12,14,17,26–28] on the outcome of LC. This is because most of the patients have undergone ultrasound examination before being referred to our hospital thus precluding inclusion of ultrasound findings in the data analysis.

**Table 5:** Literature review: Comparison between male gender and acute cholecystitis as risk factors for conversion of laparoscopic to open cholecystectomy based on multivariate logistic regression analysis

Authors^(Ref no)^	Fried *et al*.[8] 1994	Sanabria *et al*.[10] 1994	Livingstone and Rege[15] 2004	Ibrahim *et al*.[20] 2006	AI-Mulhim (present study) 2008
Male gender					
OR	2.539	4.01	1.65	1.3	0.37
95% Cl	1.477-4.366	1.76-9.09	1.62-1.67	1.02-1.96	0.03-4.24
Acute cholecystitis					
OR	5.786	12.55	3.64	1.6	1.1
95% Cl	3.21-10.43	4.55-36.18	3.59-3.70	1.2-2.01	0.86-14.08

OR: Odds ratio, Cl: Confidence interval

In our series, the lack of significant effect of gender on the rates of conversion and complications as well as operation time may be explained by (1) the relative young age of the patients, (2) a low incidence of obesity, (3) a low incidence of previous upper abdominal surgery, (4) the policy of early LC for AC, and (5) the uniform approach of an experienced surgeon. All these factors are potential effect modifiers for the outcome of LC in our study. We propose that gender differences in comorbid diseases (***P*** = 0.031) in this study may explain the longer postoperative stay in men than women (***P*** = 0.001).

## CONCLUSIONS

At a time of financial constraints and increasing demand for day-case LCs, there is an urgent need to identify the risk factors for the outcome of LC in our setting. Another aspect of accurate risk stratification is the institutional responsibilty to trainees who must be entrusted with patients in whom LC can be performed safely even on a day-case basis. This study showed that male gender was not a risk factor for conversion and complications of LC when performed by an experienced surgeon. The results also support the findings of several studies that AC, rather than male gender, is the most significant risk factor for the poor outcome of LC.
